# Assessing Optimal Cell Counts in Sperm Shape Abnormality Assays in Rodents

**DOI:** 10.3390/ani13213324

**Published:** 2023-10-26

**Authors:** Elizandra Cardoso, Maria da Luz Mathias, Rita I. Monarca, Sofia I. Gabriel

**Affiliations:** 1 CESAM—Centro de Estudos do Ambiente e do Mar, Departamento de Biologia Animal, Faculdade de Ciências da Universidade de Lisboa, Campo Grande, 1749-016 Lisboa, Portugal; fc53795@alunos.ciencias.ulisboa.pt (E.C.); mlmathias@ciencias.ulisboa.pt (M.d.L.M.); rimonarca@ciencias.ulisboa.pt (R.I.M.); 2 Departamento de Biologia da Universidade de Aveiro, Campus Universitário de Santiago, 3810-193 Aveiro, Portugal

**Keywords:** wild rodents, lab rodents, sperm abnormalities, standardization, genotoxicity

## Abstract

**Simple Summary:**

The analysis of sperm shape abnormalities is commonly used to assess the toxicity of pollutants and drugs and their genotoxic effects. This methodology is widely used based on counting the number of abnormal spermatozoa; however, in the literature, there is a wide variety of sperm counts, and standardization for cost-effectiveness and robust results remains essential. In this study, we reviewed the literature on the number of sperm counts in such assessments involving rodents, published from 1969 to 2023. A new dataset involving the analysis of two wild rodent populations was produced to infer the number of counts that provides the highest robustness of assay results. A range of 100 to 6000 was recorded in the literature review, and for each animal in the populations herein studied, 300, 500, 1000, and 2000 cells were counted sequentially, and anomalies were recorded. We propose that studies addressing sperm shape abnormalities should standardize counts to an optimal value of 1000 cells per animal, ensuring statistical power and better cost-effectiveness.

**Abstract:**

Rodents have been the preferred models for the evaluation of the toxicity of pollutants and drugs and their genotoxic effects, including sperm shape abnormalities. The scientific literature is dominated by studies conducted with model animals in laboratory conditions, but a generally accepted and standardized protocol addressing the optimal number of sperm cells to count is still lacking. In this study, we reviewed the literature regarding the number of counted sperm cells in such assessments, published from 1969 to 2023. To infer the number of counts providing the best cost/benefit regarding the robustness of the assay results, a new dataset involving the analysis of two populations of wild rodents was produced. We evaluated the frequency of sperm shape abnormalities in a total of 50 wild brown rats (*Rattus norvegicus*) captured in two port cities, aiming to detect the impact of differential sperm cell counts in the obtained results. During necropsy, the fresh epididymis tail of adult male rats was excised, and sperm cells were fixated in slides. For each animal, a total of 300, 500, 1000, and 2000 cells were sequentially counted, and head abnormalities were registered. Counting 300 sperm cells failed to detect significant differences between groups and 500 counts resulted in marginally significant differences. Only when 1000 or 2000 sperm cells were counted, significant differences emerged between groups. We propose that studies addressing sperm shape abnormalities should standardize counts to an optimal value of 1000 cells per animal, warranting robust statistical results while providing the best compromise concerning labor time.

## 1. Introduction

The toxic effects of pollutants and drug agents in organisms can be evaluated by genotoxicity studies [1]. Different approaches are used to determine their potential for inducing toxic, mutagenic, carcinogenic, reproductive, and other adverse effects on organisms [2,3,4]. Analyses of sister-chromatid exchange [5], micronucleus assay [6], bone marrow chromosome aberrations [7], and DNA damage and sperm abnormalities assay [8], constitute the most common approaches to assess hazard effects on genetic material. In animals, reproductive cells provide the first line of evidence of mutagenic alterations as a result of teratogenic effects in the organism [9,10,11].

Studies involving small non-volant mammals [12,13,14,15,16], birds [17], and fish [18], have evaluated sperm abnormalities while assessing the toxic effects of organic compounds and heavy metals, in both altered natural environments and controlled laboratory studies. Reproductive cells can be used to diagnose mutagenic alterations, including negative environmental pressure in a population [19]. This assay analyzes the frequency of occurrence of abnormal sperm cells, by assessing morphological changes in the tail or head. This is commonly performed due to its low-cost requirements and can thus be used as an initial screener to evaluate the risk of alteration in response to teratogenic agents [20,21,22,23].

Studies investigating sperm abnormalities as a tool to evaluate the toxicity of chemicals and drugs, such as anticancer and immunosuppressants, have commonly employed animal models, particularly rodents [24,25,26]. The first protocols developed by Bruce, Furrer, and Wyrobek [20,27], date back to the 1970s. The latter has been used as a reference for cell counting numbers ever since, by establishing a counting of 1000 cells per animal. In the 1990s, specific guidelines for sperm abnormality assays for rats were proposed by Filler [28], suggesting 200–500 cells per animal. However, the literature is still not consensual, and the number of cells counted varies across studies even when testing similar chemicals and drugs [24,26,29,30].

Studies evaluating teratogenic effects on wildlife using abnormal sperm cell assessment are limited [16,31]. These works are important to evaluate the real effects of potential teratogenic agents in natural environments and consequent causes on wildlife and humans inhabiting such areas. Comparative research between wild/natural and fully controlled environments (e.g., laboratory) is crucial to establish baseline levels of contamination and genotoxic effects, as well as define protocols that are repeatable and comparable across research teams and studies. The guidelines for testing performance of the male and female reproductive systems, and developmental toxicity, published by the Organization for Economic Cooperation and Development (OECD) [32], recommends counting a minimum of 200 cells. Tentativeness to establish a standard methodology, so far, has resulted in no universally accepted protocol, and highly variable counting criteria, arbitrarily defined by samplers [15,33,34,35,36]. Technological emergence led to the development of automatic cell counting equipment that became popular and gained importance [37,38]. This has contributed to increasing the standardization of protocols; however, their elevated price limits the access to such resources for a significant number of researchers in genotoxicity.

In this study, we reviewed the literature to evaluate the protocols of sperm abnormality assays, in particular the number of cells counted. We analyzed the evolution of such measurements since 1969, along with the robustness of the results provided.

To validate our results, we studied two populations of wild brown rats (*Rattus norvegicus*) inhabiting two port cities, Lisbon and Ponta Delgada (São Miguel Island, Azores), Portugal. We hypothesized that the level of urbanization influences the level of environmental toxicity, and therefore, genotoxic effects on animal populations. Differences in the average Air Quality Index (AQI) in both study cities were used as proxies of environmental exposure to pollutants. Moreover, we aimed to compare different cell counts and evaluate the cost/benefit ratio and the robustness of the data, enabling safeguards, mainly, in studies carried out in wild environments. This study handles information for researchers to make informed decisions when designing genotoxic studies.

## 2. Materials and Methods

### 2.1. Ethics Statement

This study was carried out at the Functional Biology Laboratory at the Faculty of Sciences, University of Lisbon, and was approved by the Animal Welfare Body at FCUL—ORBEA (approval number 04/2018, 12 December 2018).

### 2.2. Literature Review

Using multidisciplinary research engines Web of Science (WoS), Scopus, and Google Scholar, we compiled studies using sperm shape abnormality assay in rodent species, by manual (non-automated) approaches, observed with a microscope. Review papers were excluded, as well as publications on reproductive improvement and with unclear methodological information. The keywords used for the search were “sperm abnormality assay”, “sperm abnormalities”, “sperm morphology”, and “genotoxicity”. For each selected study, several parameters were recorded: (i) number of sperm cell counts per individual; (ii) publication year; (iii) study’s country of origin; (iv) model species; and (v) type of environment (lab versus wild). Publications not clearly stating the number of sperm cells counted or reporting methods to previous publications were removed from the analysis.

### 2.3. Sampling of Wild Rodents

Animals were obtained in the scope of a project (PTDC/SAU-PUB/29254/2017) involving the live-trapping of brown rats (*Rattus norvegicus*) in two port cities, Lisbon and Ponta Delgada, Portugal. These cities differ in their level of urbanization and therefore, levels of air pollution, as determined by the European Environmental Agency (Average Annual Air Quality Index: Lisbon—Fair; Ponta Delgada—Good). Trapping involved baited Tomahawk traps, distributed along a 10 km radius from both cities’ ports. A total of 50 adult male brown rats were used in this study (16 from Lisbon, 34 from Ponta Delgada). After capture, the animals were transported to the laboratory and euthanized by intoxication with isoflurane, in accordance with international guidelines.

### 2.4. Sperm Shape Abnormality Assay

During the necropsy, the fresh epididymis were excised from both testes. Extraction and analysis of sperm cells followed protocols adapted from Wyrobeck et al. [21] and Tapisso et al. [15]. Briefly, the cauda epididymis of rats was excised, placed in 5 mL of Sorensen buffer (pH 7.0), and centrifuged at 800 rpm for 1 min to obtain a pellet while preventing cell damage. After the removal of the supernatant, the pellet was resuspended in 5 mL of Sorensen buffer. A single drop of the suspension was transferred to a clean slide and smeared. Slides were then air-dried and fixed in absolute methanol for 10 min. After drying overnight, the slides were stained with 10% Giemsa for 1 h and coded anonymously to prevent bias during the analysis. Sperm cells were observed under an optical microscope using the 100× objective lens and the 10× ocular lens, for a total magnification of 1000×. Sperm cells were assessed for morphological head abnormalities, including wide acrosome, hook absence, short hook, and other head modifications, and grouped in eight different classes [39,40] (see Table 1 for details). For each animal, a total of 2000 sperm cells were counted, but the number and type of abnormal cells were sequentially registered after 300, 500, and 1000 cell counts.

### 2.5. Data Analyses

Differences between the number of sperm abnormalities detected in 300, 500, 1000, and 2000 sperm cell counts were evaluated by Mann–Whitney U tests per sampling location. We estimated the required sample size using a cumulative negative binomial distribution [41], considering two cell types (normal and abnormal), a minimum cell number of 10, and a power of 95%. Four probability levels of abnormality occurrence were considered: 0.01, 0.02, 0.05, and 0.1.

A generalized linear model was fitted using lmer function from the package lme4 [42] in the R software (R version 4.0.4) to test the effect of the number of cells counted on the detection of classes of abnormal sperm cells. Sampling location, number of counted cells and total number of abnormal cells detected were included as fixed factors, and individual as a random factor to account for repeated measures.

## 3. Results

### 3.1. Literature Review

**Figure 1:**
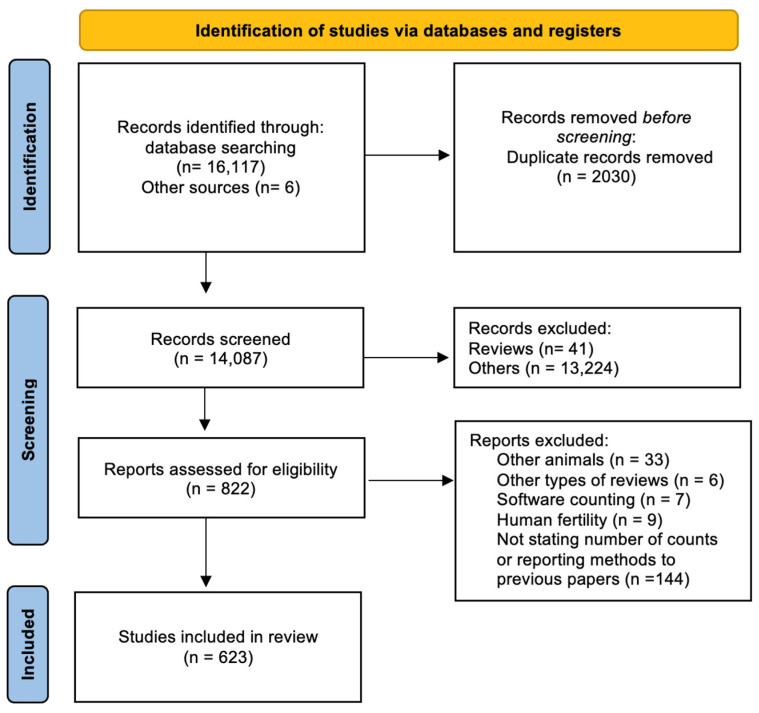
Flowchart illustrating the literature selection and review process.

**Figure 2:**
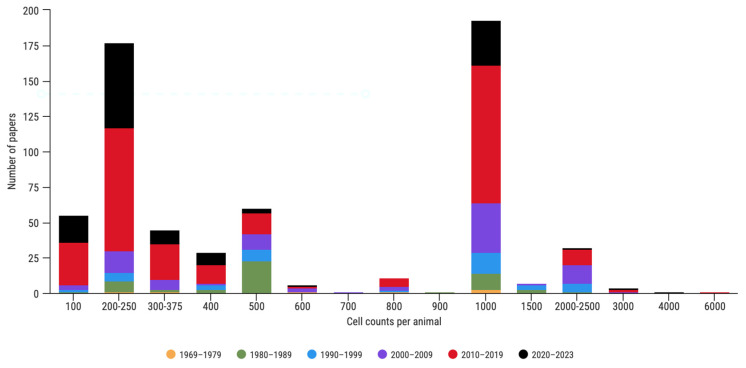
Evolution of the number of cells counted in published papers, by decade, between 1969 and June 2023, to assess genotoxic effects in rodents.

We compiled a total of 623 papers using sperm abnormalities as biomarkers of reproductive health in rodents (Figure 1), published between 1969 and June 2023. In the analyzed literature, the number of cell counts exhibited a tremendous variation, ranging from 100 to 6000 cells. The majority of publications thus far either counted 200–250 or 1000 cells during the sperm abnormality assay (28.4% and 31.3%, respectively, Figure 2). A total of 54 publications counted only 100 sperm cells per animal, all of which (except one) were published after the first OECD guidelines, which recommended at least 200 cell counts per animal [43]. Among the publications with 200 counts, 161 were published during the last two decades. Since 2010, the number of cells counted generally increased, although the most frequently used number was still 200 (34.2%). Across the years, the number of performed counts was not uniform, and in the early 2000s, counts of 1000 cells became popular (41.3%). 

**Figure 3:**
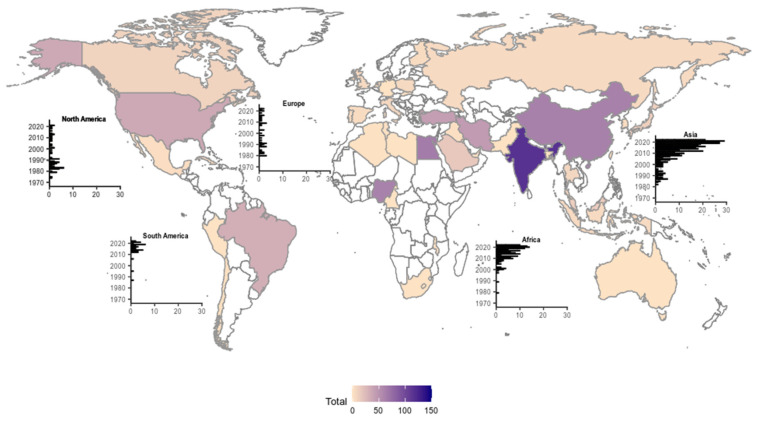
World heat map showing the sum of publications originated in each country [1969–June 2023]. Bar plots illustrate the distribution of publications through the years in each continent. Note: Oceania is not included in this temporal analysis because a single publication originated from this continent.

The analysis was extended to the regions of origin of the reviewed papers (Figure 3). The Asian continent prevailed quantitatively (headed by India), followed by Africa (headed by Egypt and Nigeria). Research papers compiled between the 1970s and 1980s were dominated by publications from the European and North American continents with the onset of genotoxic studies in the late 1960s and early 1970s. We also identified a trend in the number of cells counted in papers originated within the same country or region (e.g., 800 counted cells, see supplementary data). Over the years, this technique has gradually been abandoned in Europe and North America and recent inputs are dominated by Asian and African publications.

### 3.2. Sperm Shape Abnormalities–Case-Study with Wild Rodents

**Figure 4:**
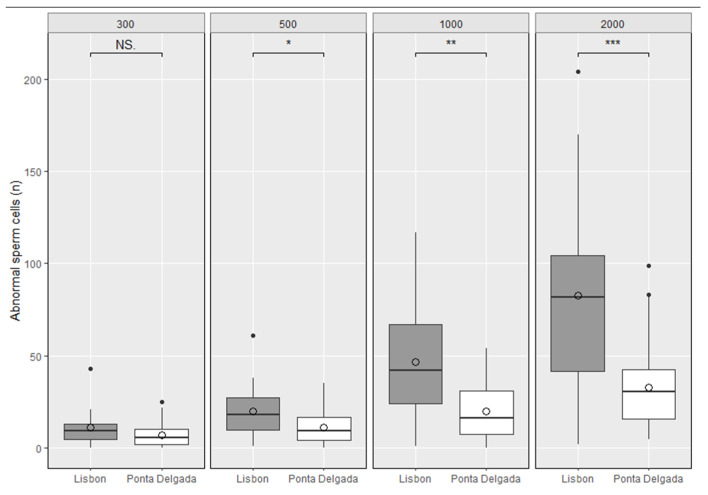
Number of abnormal sperm cells detected per animal in 300, 500, 1000, and 2000 cell counts, in *Rattus norvegicus* from Lisbon and Ponta Delgada; Mann–Whitney U test: NS. non-significant, * *p* ≤ 0.05; ** *p* ≤ 0.01; *** *p* ≤ 0.001).

The results obtained in the samples collected in Lisbon and Ponta Delgada are shown in Figure 4. Overall, *R. norvegicus* from Lisbon exhibited more sperm abnormalities than individuals from Ponta Delgada. Differences between sperm abnormalities detected in 300 sperm counting were not statistically significant (*p*-value = 0.139), for the 500 sperm counting presented some significance (*p*-value = 0.040), and the 1000 and 2000 sperm counting were statistically significant (*p*-value = 0.002 and *p*-value < 0.001, respectively). Table 2 shows the number of cells needed to detect 10 abnormal cells, considering different probabilities of occurrence.

The analysis of abnormal cells per class showed that the most frequently observed class of abnormal cells is the short hook/banana, followed by a triangular shape (in Lisbon) and hook with wrong angle (in Ponta Delgada). The number of classes of abnormal cells identified were highly influenced by the number of counted cells (*t* = 7.72; *p* < 0.001) and the total number of abnormalities detected (*t* = 4.288; *p* < 0.001), while the city of origin did not have a significant effect (*t* = −0.800, *p* = 0.430). The maximum number of cell classes per animal was six, registered only when 2000 cells were counted. However, seven out of eight classes were found with the minimal number of cell counts (Table 3). 

## 4. Discussion

The trustworthiness of risk assessment studies depends on the reliability and integrity of their procedures. The development of internationally accepted guidelines, such as those proposed by, e.g., the OECD [43] is key to ensuring high-quality and robust data that accurately report hazards [44,45]. 

In this study, we inferred the most cost-effective number of cell counts in the context of assays involving manually counting sperm head anomalies in rodent models, in order to accommodate both lab and field studies and chronic and acute levels of exposure. This technique has been widely used in the context of genotoxicity assessments over the past few decades, with increasing contributions from lower-income countries since the beginning of the millennium (Figure 3). Thus, defining an optimal number of counts that simultaneously ensures robust statistical power while avoiding unnecessary laborious efforts is key, given that such a methodology is extremely time-consuming. A reduced number of counts may be insufficient to uncover statistically significant differences between study groups, particularly when data variability is high, and contamination levels are reduced. On the other hand, an excessive number of cell counts demands unnecessary operational effort without a proportional increase in statistical robustness.

As early as 1975, Wyrobeck and Bruce [20] published the first protocol proposing 1000 sperm cell counts per animal. However, it was not until the first decade of the 2000s that it became more frequently cited and followed by others [30,46,47]. In subsequent years, high variability in the number of cell counts was still observed as protocols used in genotoxic assays became common in pharmaceutical assays [see supplementary material for details].

Over the years, the number of cell counts in sperm abnormality assays involving rodents has widely varied, ranging from 100 [48,49,50,51,52] to 6000 [53]. Attempts to incorporate some level of standardization in protocols involving sperm abnormality tests under laboratory settings, such as the OECD guidelines [32], and the Filler publication [28], were successful at some point establishing 200 and 200–500 cell counts, respectively. The publications that follow the recommendations of Wyrobeck and Bruce [20] continue to be the majority. However, very recent publications (2019–2023) following protocols considering only 100 cell counts per animal are still being published [54,55,56,57,58].

Concerns about the statistical power of genotoxicity data have been around since the mid-1990s, when Seed et al. [59] proposed methods to assess the motility, morphology, and sperm counts in rats, rabbits, and dogs. The study suggested that more investment should be made towards statistical analyses involving sperm morphology but did not specifically mention an optimal number of cell counts regarding the analyses of abnormality frequencies.

Overall, sperm morphology assays remain valid, with a higher and increasing prevalence in African and Southern Asian countries (e.g., India, Egypt, and Nigeria, Figure 3). One possible factor influencing this distribution could be the resources allocated to scientific research in these regions [60,61]. It is plausible that in developed countries (as listed by the United Nations), these methods were more likely replaced by automated scanning techniques, although they are still widely used in many other regions of the world [62,63]. Given the search parameters for our literature review and exclusion rules, our results are unable to support the former. In laboratory settings, the genotoxic effects of a certain agent may vary depending on the level of toxicity of the tested agent. In our literature compilation (see Supplementary Materials), we observed several studies reporting a multiplicity of cell counts when testing the effects of similar xenobiotics [24,26,29,30]. As such, we highlight the importance of standardizing the number of cell counts per animal in genotoxicity assessment protocols involving the determination of sperm abnormality frequencies, ensuring that results obtained are robust and comparable. Our study assumes that all the other protocol steps are optimized, not attempting to evaluate other methodological discrepancies between studies.

In the case study presented herein, we observed that the number of sperm cells counted per animal was crucial for the interpretation of the obtained results, either detecting or not detecting statistically significant differences between groups. When counting 300 sperm cells, no statistically significant differences were observed between the two rat groups (*p* = 0.139), while counting 500 cells unveiled marginally significant differences (*p* = 0.04). Only when 1000 cells were counted did a highly robust difference emerge between groups (*p* = 0.002), which was even higher for the 2000 cell counts (*p* = 0.0006). Although in laboratory-based studies, inter-individual variance is reduced, and environmental factors highly controlled, in studies involving wild-caught animals, individuals are subject to various biotic and abiotic constraints (that contribute to data variance). Among these restrictions, we highlight the putative influence of wild animals’ age, known to increase levels of sperm abnormalities [64]. Within our dataset of commensal wild brown rats, we did not observe an impact of aged specimens, given that in commensal populations the lifespan is usually shorter than in laboratory animals [65,66] due to predation, intra-specific competition, diseases and pest management control. The genetic variability of wild populations is key for understanding ranges of response to xenobiotics and induced damages [67]. The use of highly inbred stocks can result in stable results within the testing animals, but elevated levels of false results when applied to other strains/stocks [68]. In our case study, differences were observed even at the individual level, depending on the number of cells counted: 300, 500, 1000, or 2000. The percentage of abnormal cells observed among each studied population varied from 3.7% (at 300 cell counts) to 4.7% (at 1000 cell counts) in Lisbon and from 1.7% (at 2000 cell counts) to 2.4% (at 300 cell counts) in Ponta Delgada (see Table 3). Also, there was no pattern regarding which number of cell counts produced the highest or lowest percentage of detected abnormalities. Due to this stochasticity, it is important to account for variations in density of abnormal cells throughout the slide. By counting a larger number of cells, this effect will likely have a lower impact, portraying a better representation of each animal’s cell abnormalities. The biological relevance of sperm abnormalities has been demonstrated in rodents at levels as low as 1% (e.g., [69]), by reducing fertility levels [70]. Abnormally shaped sperm cells exhibit DNA damage, express chromatin and cytoskeletal alterations [71,72], becoming unlikely to be selected within the female genital tract [73], compromising fertility [74].

Our power analysis suggests that previous knowledge of the expected genotoxic influence may lead to an optimal choice of cell counting. However, in most scenarios, such effects are unknown and, thus, unpredictable, in particular when assessing genotoxic effects on natural environments, with unknown levels of contamination or exposure. Considering the total sperm abnormalities observed in our case study, between ~2% in Ponta Delgada and ~4% in Lisbon, we validate the choice of 1000 cell counts per animal to accommodate different levels of contamination scenarios as calculated by the power analysis (Table 2). Robust datasets are critical to unravel genotoxic effects in wild environments. Therefore, it is pivotal that the number of cells examined is large enough to be representative of the functional reproductive cells allowing the detection of abnormalities in each study group. If statistically significant differences exist between experimental groups, the total number of cells counted must be sufficient to uncover them. The effects of exposure to contaminants can be highly variable in terms of concentration, time of exposure and nature of the xenochemical (e.g., [46,70]). The rates of expected effects on wild populations are difficult to predict, because in most cases the factors mentioned above are unknown. Thus, when assessing wild populations, environmental conditions are a key point. Moreover, interactions between genotoxic agents should not be disregarded [75].

This case study shows that, in a dataset obtained from wild-caught rodents inhabiting moderately polluted urbanized cities [76], 300 cell counts were insufficient to detect statistically significant differences between the analyzed groups, and, therefore, genotoxic effects due to environmental causes. Other studies using wild animals support these observations by failing to detect significant alterations in the sperm morphology [31,77,78]. The latter has particular interest when sperm morphology is the only tool to assess genotoxic effects. Often, null results are blinded by effects highlighted by other methods.

An optimal number of cell counts is not only relevant concerning the total number of sperm anomalies detected in study groups but also regarding the type of anomalies identified. To our knowledge, different types of anomalies have not been connected with sperm cell activity, as all are potentially hazardous and compromise sperm function. However, we noticed that rarer anomalies may become undetected if a smaller number of cell counts is considered. This was the case for three out of eight classes of observed sperm abnormalities among our dataset. When only 300 cells were counted, no amorphous sperm heads were detected among the Lisbon rat population, nor swollen acrosomes or swollen hooks among the Ponta Delgada population (Table 3).

Our literature compilation returned only seven publications using wild rodent populations as bioindicators of environmental pollution. The outcomes of these publications have a wide range of cell counts: 100 cells [31], 200 cells [77,78], 1000 cells [16,79], and 1500 cells [19,80].

We suggest that studies addressing wild animals in natural conditions, by standardizing cell counts to 1000, would provide sufficiently robust and repeatable results, allowing existing statistical differences to emerge, considering frequencies of abnormalities occurrence below 2%. Standardizing cell counts to 1000 would also be the most cost-effective option as cell counts > 1000 severely stretches the analytical time with no improved return. We also consider that increasing the cell pool by counting elevated numbers of cells from single animals should be avoided to account for inter-individual variation. 

When compared to those in laboratory settings, genotoxicity studies in natural environments are rare. The study of reproductive parameters in wild species is key not only in toxicology assessments but as a proxy of overall health of wild populations [81]. Knowledge of sperm traits can be informative of reduced reproduction rates in wild populations and even decline in endangered species. This information, applied to other animal groups, could lead to improvements in the success of conservation programs [82] and environmental restoration.

## 5. Conclusions

This review highlights the need for an optimized approach regarding research on genotoxicity assessment involving (manual) counts of sperm head abnormalities in rodents. By reviewing the scientific literature and using a dataset from wild-caught rodents as a case-study, we concluded that counting 1000 sperm cells per animal constitutes the optimal trade-off between statistical robustness and operational costs. If a common and optimized methodology is followed by most researchers, the obtained results of genotoxic assessments will be comparable, even inter-laboratories, which is currently not the case.

## Figures and Tables

**Table 1:** Description of morphological sperm head abnormalities identified in the smears of *Rattus norvegicus* epididymal cauda (×1000), pictures using Giemsa stain.

Class	Description	Picture
Normal sperm	Head accented by a marked hook, leading to a comma-like form.	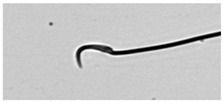
Short hook/Banana	Shortening of the hook, leading to a banana-like form.	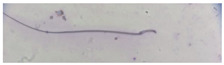
Hook at wrong angle	Shows a crooked hook.	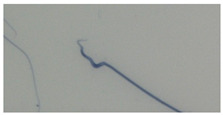
Straight/no hook	Similar to a straight line.	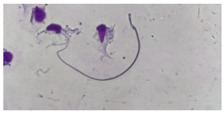
Triangular	Similar to a triangle/Arrow.	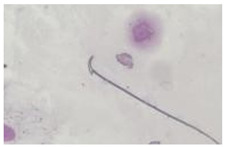
Amorphous	Altered and indefinite form of the sperm head.	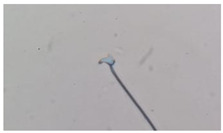
Swollen acrosome	Enlargement of the basal area of the sperm head.	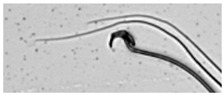
Acute curvature	Pronounced hook.	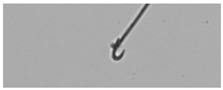
Swollen hook	Enlargement of the hook tip.	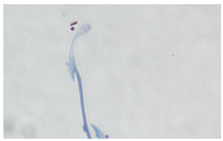

**Table 2:** Number of required cell counts necessary to detect 10 abnormal cells, with a power of 95% and 99%, with different levels of probability of occurrence.

Probability of Abnormality	Number of Cell Counts Needed	
	Power 95%	Power 99%
0.01	1693	1985
0.02	848	986
0.05	329	387
0.1	158	221

**Table 3:** Distribution of sperm abnormality classes per number of counted cells in Lisbon and Ponta Delgada. *n*–sum of abnormal cells found in the total analyzed animals by location.

		Sperm Abnormality (Total/Classes)																	
		Total Abnormalities (All Individuals)		Short Hook/ Banana		Hook Angle		Straight Hook		Triangular		Amorphous		Swollen Acrosome		Acute Curvature		Swollen Hook	
	Cell Counts	*n*	%	*n*	%	*n*	%	*n*	%	*n*	%	*n*	%	*n*	%	*n*	%	*n*	%
Lisbon (*n* = 16)	300	177	3.7	144	81.4	8	4.5	1	0.6	18	10.2	0	0.0	1	0.6	4	2.3	1	0.6
	500	323	4.0	255	78.9	18	5.6	1	0.3	33	10.2	2	0.6	2	0.6	8	2.5	4	1.2
	1000	751	4.7	577	76.8	49	6.5	1	0.1	85	11.3	5	0.7	3	0.4	23	3.1	8	1.1
	2000	1319	4.1	996	75.5	94	7.1	4	0.3	165	12.5	8	0.6	3	0.2	41	3.1	8	0.6
Ponta Delgada (*n* = 34)	300	246	2.4	191	77.6	41	16.7	2	0.8	4	1.6	0	0.0	0	0.0	8	3.3	0	0.0
	500	396	2.3	304	76.8	70	17.7	5	1.3	5	1.3	0	0.0	1	0.3	10	2.5	1	0.3
	1000	684	2.0	530	77.5	109	15.9	11	1.6	12	1.8	0	0.0	1	0.1	18	2.6	3	0.4
	2000	1141	1.7	899	78.8	152	13.3	24	2.1	23	2.0	2	0.2	1	0.1	34	3.0	6	0.5

## Supplementary Materials

The following supporting information can be downloaded at: https://www.mdpi.com/article/10.3390/ani13213324/s1, Sheet S1: References of the review.
